# Effects of hypertension in patients receiving mechanical thrombectomy: A meta-analysis

**DOI:** 10.1097/MD.0000000000019803

**Published:** 2020-04-17

**Authors:** Zhengzhou Yuan, Ning Chen, Muke Zhou, Jian Guo, Yanan Zhang, Yanbo Li, Li He

**Affiliations:** a Department of Neurology, West China Hospital of Sichuan University, ChengDu; b Department of Neurology, Affiliated Hospital of Southwest Medical University, LuZhou, China.

**Keywords:** acute ischaemic stroke, blood pressure, hypertension, mechanical thrombectomy, meta-analysis

## Abstract

**Background:**

Available evidence shows conflicting results with regard to a potential detrimental effect of hypertension on clinical outcomes in patients who undergo mechanical thrombectomy (MT). We performed a meta-analysis to evaluate the impact of hypertension on the prognosis of patients with acute ischaemic stroke (AIS) treated by MT.

**Methods:**

We systematically reviewed previous studies in the PubMed, EMBASE, and Cochrane library databases that reported MT outcomes in AIS patients and their relationships with hypertension or blood pressure. We used a poor outcome (modified Rankin score >2 at 3 months) as the primary end point. Mortality and symptomatic intracranial hemorrhage were the secondary end points. We incorporated a random effect for trials in all models.

**Results:**

Data from 6650 patients in 31 articles that evaluated the effect of hypertension or blood pressure on outcomes after MT were included. Compared with patients without hypertension, patients with hypertension had significantly higher odds of a poor outcome (odds ratio 0.70; 95% confidence interval 0.57–0.85; *I*^2^ = 43%) and higher mortality (odds ratio 1.70; 95% confidence interval 1.26–2.29; *I*^2^ = 33%). Symptomatic intracranial hemorrhage did not differ by patient hypertension status.

**Conclusions:**

The present study confirms that hypertension and high blood pressure are associated with a poor outcome at 3 months after MT in AIS patients. However, the causal relationship between hypertension and a poor outcome remains undetermined, and further investigations are required to ascertain whether AIS patients receiving MT could benefit from intensive blood pressure control.

## Abstract 2

Supplemental Digital Content is available in the text

## 1 Introduction

Stroke is the second-leading cause of death in the world and is a major cause of serious disability for adults.^[1]^ Hypertension is the leading contributor to overall mortality and the third-leading cause of lost healthy life years in stroke patients worldwide.^[2]^ Ischaemic stroke contributes to 70% to 80% of all stroke cases. Several randomized clinical trials have demonstrated the safety and efficiency of mechanical thrombectomy (MT) in the management of acute ischaemic stroke (AIS) caused by larger vessel occlusion. Although significant improvements have been demonstrated by recent randomized clinical trials, many patients do not have good functional outcomes even though timely and successful revascularization is achieved.^[3]^

Previous studies have shown that hypertension is related to poor functional outcomes, mortality and haemorrhagic transformation after intravenous or intra-arterial thrombolysis.^[4,5]^ It is speculated that hypertension may also be associated with the treatment efficiency of MT, and several post hoc trials have indicated that in patients treated with MT, hypertension was independently associated with poor outcomes at 3 months.^[6,7]^ In contrast, other studies showed that there was no significant difference in 90-day independent functional outcomes and complications between the hypertension group and the nonhypertension group. Available evidence shows conflicting results with regard to a potential detrimental effect of hypertension on clinical outcomes. Therefore, we aimed to conduct a meta-analysis of published studies to determine whether hypertension or admission blood pressure was associated with a poor outcome in patients treated with MT for AIS.

## 2 Methods

The systematic review and meta-analysis were performed according to the preferred reporting items for systematic reviews and meta-analyses statement.^[8]^ The common evidence-based medicine framework Patient/Population, Intervention/Exposure, Control, Outcome was used to specify our research question: Did patients with AIS caused by emergent large vessel occlusion who received MT (patient population) accompanied with hypertension (exposure) have a poor functional outcome, higher rates of mortality and symptomatic intracerebral hemorrhage (sICH) (outcomes) than did patients without hypertension (control)?

### 2.1 Ethical review

The meta-analysis data was from published research studies. Hence, ethical review is not applicable.

### 2.2 Search strategy

We searched studies without language restrictions from the MEDLINE, EMBASE, and Cochrane library databases from January 2010 to November 2018 to identify potentially relevant studies reporting the influence of hypertension or blood pressure on efficacy or safety outcomes in AIS patients treated with MT. Search terms were set with the subject headings and keywords “Stroke or Brain Infarction or Cerebral Infarction or Cerebrovascular Disorders,” “Thrombectomy or Mechanical Thrombolysis or Endovascular Treatment or Endovascular Therapy,” and “Hypertension or Blood Pressure.”

### 2.3 Study selection

**Figure 1:**
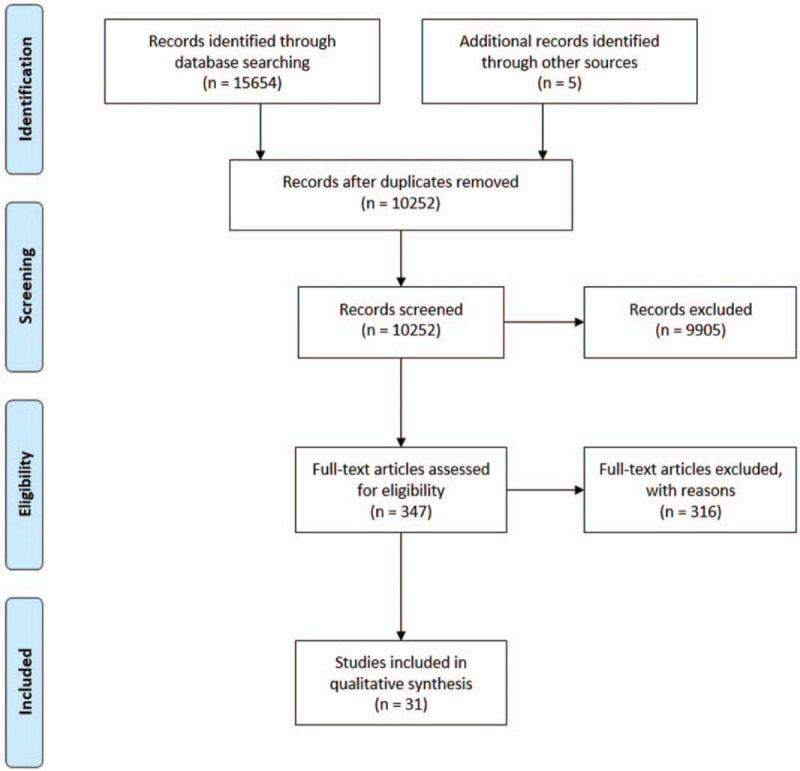
PRISMA flow chart of study selection process. PRISMA = preferred reporting items for systematic reviews and meta-analyses.

Two qualified investigators (Zhengzhou Yuan and Ning Chen) independently screened titles and abstracts of references identified by the searches. In cases of disagreement, consensus was achieved through referral to a third reviewer (Muke Zhou). After primary selection, the full text of closely related studies was obtained and re-evaluated for eligibility, which determined the final inclusion of the study in the meta-analysis (Fig. 1). Studies were included if they satisfied the following criteria:

(1) the age of included AIS patients was ≥18 years old, and the sample size was ≥30;
(2) all included patients were treated with MT; and
(3) a statistical analysis of the association of outcomes (efficacy or safety) with a history of hypertension or blood pressure was reported.

Hypertension was identified from self-reports or doctors’ measurements at baseline and follow-up measures or questionnaires meeting at least 1 of 3 JNC8^[9]^ criteria: use of antihypertensive medicines, systolic blood pressure (SBP) ≥140 mm Hg, or diastolic blood pressure (DBP) ≥90 mm Hg. Only those that were published as full-length articles were considered. We excluded case reports, nonhuman studies, meta-analyses, guidelines, technical notes, or studies with treatment time >24 hours after stroke onset. A study pertaining to posterior circulation AIS was excluded. For studies with overlapping published data from the same institution, the most updated or most inclusive data were chosen for the analysis.

### 2.4 Data extraction and quality assessment

Data extraction from eligible studies was also performed by 2 investigators (Jian Guo and Yanan Zhang) with a standardized data extraction form that included the first author, publication year, country included, study period, study design, number of participants, participants’ age and sex, occlusion sites, National Institutes of Health Stroke Scale [NIHSS] score at admission, MT devices used (stent retriever, Merci retriever, Penumbra system, or aspiration), definition and incidence of outcomes (functional outcomes, mortality, sICH), and associations with hypertension or blood pressure (maximum systolic blood pressure or diastolic pressure during the first 24 hours following MT) on functional outcomes. The quality of the articles was assessed using the Newcastle–Ottawa quality assessment scale. All the articles fulfilling the inclusion criteria were included in the meta-analysis independently of the quality score.

### 2.5 Outcome variables and statistical analysis

The primary end point of the present study was a poor outcome, defined as a modified Rankin scale score (mRS) of 3 to 6 at 90 days after MT treatment. The second outcome included sICH (defined according to definitions in the original studies) and mortality at 90-day follow-up.

Using patients without a history of hypertension as the reference group, the effect size for hypertension according to the odds ratio (ORs) and associated 95% confidence interval (CIs) was calculated by the Mantel–Haenszel method. The effect sizes for maximum SBP and DBP during the first 24 hours following MT were reported as the mean difference based on the inverse variance method between patients with and without poor outcomes. Heterogeneity between studies was quantified with the *I*^2^ statistic (with ≥50% indicating substantial heterogeneity). We performed random-effects meta-analyses to pool the estimates for each comparison. We performed subgroup analysis to investigate the primary end point. We also performed a sensitivity analysis examining the comparative outcomes only including multi-center studies. Funnel plots were used to assess publication bias. Meta-analysis and meta-regression were conducted using Review Manager 5.3. A probability value of <0.05 was considered significant. Except for heterogeneity testing, the significance was accepted at a probability value of 0.10.

## 3 Results

### 3.1 Study selection and characteristics

A total of 15,659 articles were found in the initial literature search, and 5402 records were duplicated. After the records were reviewed at the title or abstract level, 9905 records were eliminated: 6513 studies not related to our study; 2732 reviews, errata, comments, case reports, or study protocols; and 313 studies in which the number of patients was <30. Of the 347 remaining articles, the full text was reviewed. In total, 31 articles^[6,7,10–38]^ that included a total of 6650 patients (4174 hypertension and 2476 nonhypertension) were included in the meta-analysis. The preferred reporting items for systematic reviews and meta-analyses flow diagram is provided in Figure 1.

The proportion of hypertension patients was 62.8% (4174/6650). All studies reported the occlusion location, 18 studies included anterior circulation strokes only, and the remaining 13 studies included both anterior circulation strokes and posterior circulation strokes. Of the 23 studies that reported the correlation between hypertension and the 3-month follow-up mRS score, 11 studies were conducted in Europe, 6 in the United States, and 6 in Asia. Details of the individual trials used for analysis for each group, including methodological and baseline characteristics of the included studies, are listed in Table 1 in the Supplemental Material.

**Table 1:**
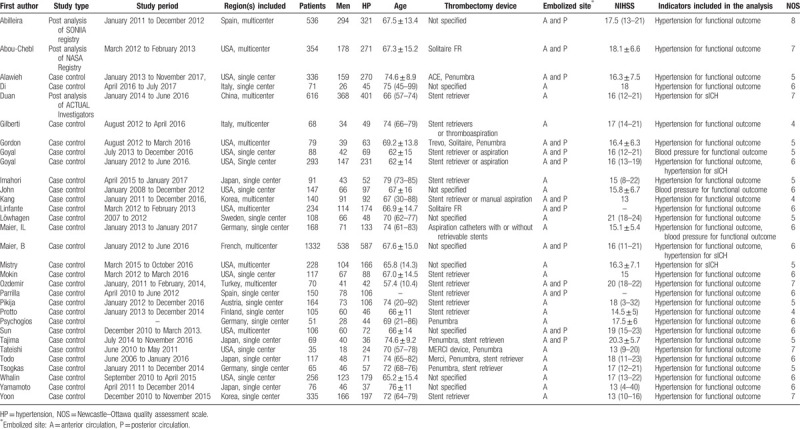
Summary of included articles assessing impact of hypertension and/or admission blood pressure on acute ischemic stroke outcomes after mechanical thrombectomy.

### 3.2 Hypertension and outcomes

**Figure 2:**
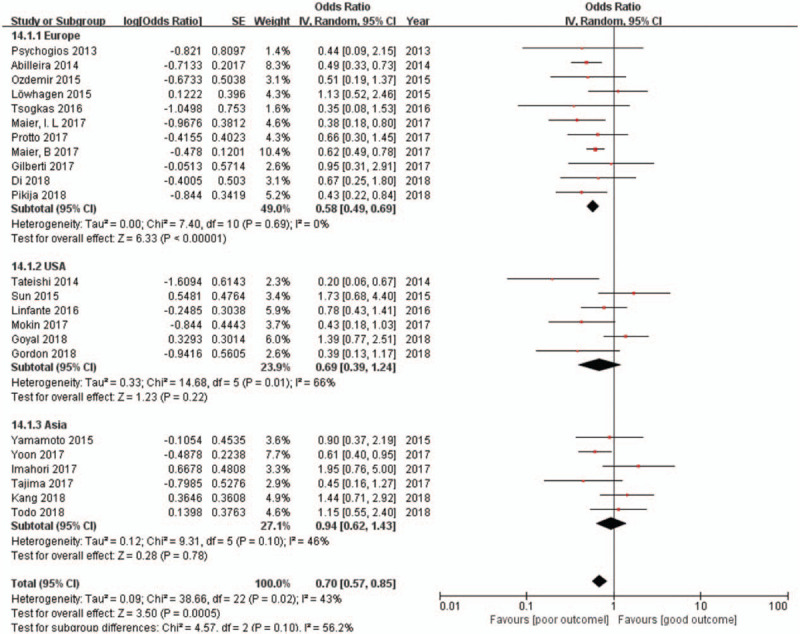
Forest plots of unadjusted ORs for poor outcome associated with hypertension. ORs = odds ratios.

The available unadjusted results of the effect of hypertension on poor outcomes were reported in 23 studies (n = 4430). Compared with those without hypertension, patients with hypertension were associated with significantly lower odds of functional independence at the 90-day follow-up (OR 0.70, 95% CI 0.57–0.85; *I*^2^ = 43%) (Fig. 2). The subgroup analysis showed that Europeans with hypertension were significantly associated with poor outcomes; however, this trend was not significant in Asians and Americans. The effect sizes of the effect of maximum SBP during the first 24 hours following MT on functional outcomes were reported in 3 studies (n = 403), and patients without functional independence had a significantly higher maximum SBP than did patients with functional independence (pooled effect size −13.72, 95% CI −18.27 to −9.16; *I*^2^ = 0%) (Fig. 5). A similar result was found in maximum DBP during the first 24 hours after MT (2 studies, n = 235; pooled effect size −7.02, 95% CI −11.72 to −2.33; *I*^2^ = 0%) (Fig. 6).

**Figure 3:**
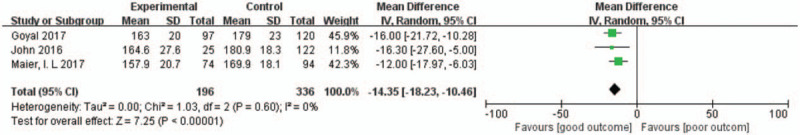
Forest plots of unadjusted ORs for mortality associated with hypertension. ORs = odds ratios.

**Figure 4:**
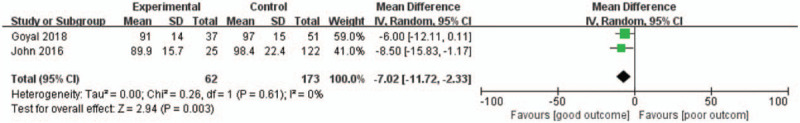
Forest plots of unadjusted ORs for sICH associated with hypertension. ORs = odds ratios, sICH = symptomatic intracranial hemorrhage.

Hypertension was also associated with higher mortality after 90-day follow-up (6 studies, n = 3001; OR 1.70, 95% CI 1.26–2.29; *I*^2^ = 33%) (Fig. 3). However, we did not find a difference in sICH (4 studies, n = 2469; OR 0.91, 95% CI 0.62–1.34; *I*^2^ = 31%) (Fig. 4) between patients with and without hypertension. Subgroup analysis found that studies only included a mean NIHSS greater than 17 points (9 studies, n = 1455; OR 0.68; 95% CI 0.48–0.96; *I*^2^ = 38%), and less than 17 points (13 studies, n = 2771; OR 0.70; 95% CI 0.52–0.93; *I*^2^ = 53%) were both associated with significantly lower odds of functional independence. The results of the multi-center studies that were included were also highly consistent (8 were multi-center studies, n = 2666; OR 0.72; 95% CI 0.53–0.97; *I*^2^ = 46%).

**Figure 5:**
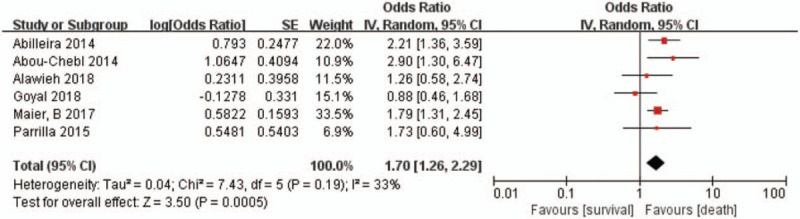
Forest plots of unadjusted MDs in maximum SBP during the first 24 h following MT between patients with and without functional independence. MDs = mean differences, MT = mechanical thrombectomy, SBP = systolic blood pressure.

**Figure 6:**
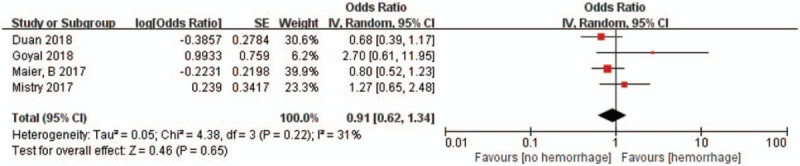
Forest plots of unadjusted MDs in maximum DBP during the first 24 h following MT between patients with and without functional independence. DBP = diastolic blood pressure, MDs = mean differences, MT = mechanical thrombectomy.

### 3.3 Publication bias

**Figure 7:**
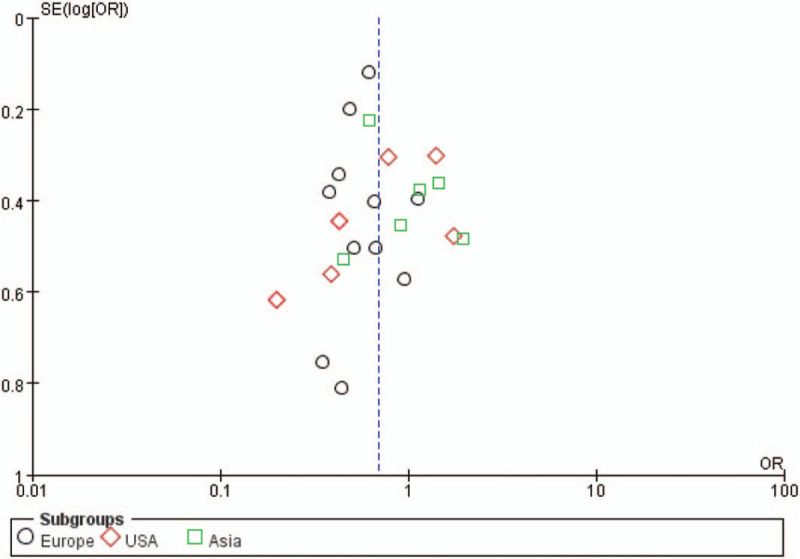
Funnel plot of the publication bias in the meta-analysis.

The funnel plot did not suggest publication bias for hypertension to poor outcomes and mortality after 3 months of follow-up (Fig. 7).

## 4 Discussion

Among all the neurologic diseases in adults, stroke ranks first in importance and frequency.^[39]^ Most strokes in the United States are ischemic. Ischemic stroke is due to occlusion of a cerebral blood vessel and causes cerebral infarction. Awakening with or experiencing the abrupt onset of focal neurologic deficits is the hallmark of the diagnosis of ischemic stroke.^[40]^ The most common presenting symptoms of ischemic stroke are unilateral weakness and speech disturbance. Atherosclerotic thrombotic disease of the cerebral or extracerebral vessels, and cerebral embolism are 2 main causes of ischemic stroke. Epidemiologic studies have established many ischemic stroke risk factors, such as age, family history, chronic kidney disease, and sleep apnea, but the most importance of these are hypertension, diabetes mellitus, atrial fibrillation, and hyperlipidemia.^[1]^ There is a well-established relationship between blood pressure and the risk for developing of ischemic stroke, the relationship is consistent, continuous, and independent of other risk factors.

Over the past 2 decades, the treatment of acute ischemic stroke has been deeply transformed. Began 20 years ago, administered Intravenous alteplase thrombolysis within 4.5 hours, increases the odds of no significant disability (mRS 0–1) by about a third.^[41]^ Since 2015, there has been clear evidence that addition of MT can further improve the outcome in patients with severe neurologic deficits from a proximal intracranial vessel occlusion.^[42]^ The addition of MT with second-generation devices to alteplase within 6 hours of ischaemic stroke doubles the rate of reperfusion at 24 hours and functional independence, and increases the likelihood of improving by 1 point or more on the mRS by 2.5 times. Candidates for MT are patients with severe neurologic symptoms (NIHSS score ≥6), no major ischemic changes on the baseline CT scan (Alberta Stroke Program Early CT Score score ≥6), good pre-stroke functional status (mRS ≤2), early presentation (Time from symptom onset to groin puncture <24 hours) and Presence of proximal intracranial artery occlusion.^[43]^

Evidence from currently available data suggests that hypertension and maximum SBP and systolic blood pressure (SDP) during the first 24 hours following MT are associated with lower rates of good functional outcomes at 3 months after MT in AIS patients; moreover, hypertension is associated with higher rates of mortality. In contrast, no difference in sICH was found between patients with and without hypertension. The subgroup analysis showed that Europeans with hypertension were significantly associated with poor functional outcomes but that Asians and Americans (especially Asians) were not. This finding may be attributable to the following explanations. First, there are differences in the genetic form and type of hypertension among Europeans, Americans, and Asians.^[44]^ Second, Asians have a higher proportion of intracranial atherosclerosis, and higher blood pressure can provide more cerebral perfusion; Third, the heterogeneity among the American studies included is greater (*I*^2^ = 63%). Finally, the sample size of the United States (n = 864) and Asia (n = 828) is smaller than that of the European studies (n = 2738).

It has been estimated that 26% of the world's adult population had hypertension in the year 2000, and the proportion will increase to 29% by 2025. The proportion of hypertension patients was 62.8% in our study.^[45]^ Numerous studies have confirmed that hypertension and high blood pressure are associated with sICH and poor functional outcome in acute ischaemic stroke,^[46,47]^ and higher maximum SBP was also associated with poor functional outcomes after MT.^[36]^ The post hoc data analysis of the Multicenter Randomized Clinical Trial of Endovascular Treatment for Acute Ischaemic Stroke in the Netherlands reported that BP did not affect the benefit or safety of MT in patients with AIS, but it also confirmed a strong correlation between systolic blood pressure and functional outcomes in ischaemic stroke, and the association was U-shaped. Both low and high baseline mean SBP measurements were associated with poor functional outcomes. They also found that higher mean SBP was associated with increased sICH risk.^[48]^ In our meta-analysis, hypertension was associated with poor outcomes but did not increase sICH risk. The proportion of patients with high systolic hypertension in this study was small, and these studies started later, when the technology was more mature.

The 2019 update of the American Heart Association/American Stroke Association early stroke management guidelines recommend the treatment of BP if it is higher than 180/105 mm Hg both during the procedure and for 24 hours afterward for patients with AIS who undergo MT; the guidelines also recommend the maintenance of BP at a level of <180/105 mm Hg if patients achieve successful reperfusion.^[43]^ However, the optimal guided blood pressure treatment data during and after MT are very limited. The ESCAPE studies suggest that SDP ≥150 mm Hg may help to promote and maintain adequate collateral blood flow when the artery is still occluded, and once reperfusion is achieved, it is a wise choice to control the patient's blood pressure within a normal range. The DAWN study recommended that SDP is maintained at a level <140 mm Hg in the first 24 hours after arterial recanalization in MT patients. A network meta-analysis also indicates that treating patients with a reduction in SBP below 140 mm Hg may significantly reduce the risk of vascular disease and all-cause mortality. These findings support more intensive SBP control among adults with hypertension.^[49]^ It is reasonable to assume that the effect of intensive SBP and SDP control would become visible in AIS patients. Although our results show that maximum SBP and SDP during the first 24 hours following MT are related to unfavorable functional outcomes, the precise association between maximum SBP and SDP with functional outcomes in AIS patients needs to be further studied.

Our meta-analysis has some limitations. First, all the studies included in our analysis were observational, and most of them cannot control for baseline characteristics. It is reported that patients with hypertension are often older and have more frequent histories of diabetes, dyslipidemia, heart failure and carotid stenosis, which indicates that the pooled unadjusted results of this study may be influenced by baseline data. However, similar results were obtained in studies in which the average NIHSS score at admission was greater than or less than 17, and the same results were also achieved in multi-center studies, which partly enhanced the reliability of our results. Second, subgroup analysis by region showed that Europeans with hypertension had poor functional outcomes, but the relationship between hypertension and poor outcomes is not statistically significant in Asia, which affects the applicability of this result to a certain extent. Third, the definition of sICH was obtained from the original study, rather than from a standardized definition, which may affect the reliability of the pooled results at this end point. Finally, because the direct comparison of blood pressure and prognosis of MT is too limited, we included some articles indirectly comparing blood pressure and prognosis, and we did not evaluate the quality of the included studies. We tried to overcome this problem through sensitivity analysis, which included an analysis of studies with high NIHSS scores at admission and subgroup analysis of studies conducted at multiple centers.

Several research questions have arisen from this work, the most important of which is how to achieve and maintain the optimal range of blood pressure control after MT, because these experiments have found that lower maximum blood pressure is related to good function outcomes; however, there is no optimal range of blood pressure control.^[50]^ Intravenous medication, even intravenous combination therapy, will be an important part of this solution. The application of non-pharmacological methods, such as weight loss and dietary sodium restriction, may be helpful for hypertension but may be insufficient for most patients with AIS who are treated with MT. Randomized trials of these patients with different blood pressure control strategies are clearly warranted, as these strategies concern a strong prognostic factor related to the poor prognosis of MT, and they also represent a few controllable risk factors for cerebrovascular disease.

In conclusion, our study revealed that hypertension, high maximum SBP and SDP are associated with poor functional outcomes at 3 months after MT in AIS patients. However, the causal relationship between hypertension and poor outcomes remains uncertain. Further larger and multi-institutional randomized controlled studies may provide important novel information for the management of AIS patients with hypertension who received MT and determine the ideal blood pressure management strategy.

## Author contributions

**Conceptualization:** Zhengzhou Yuan.

**Data curation:** Zhengzhou Yuan, Jian Guo, Yanbo Li, Li He.

**Formal analysis:** Zhengzhou Yuan, Jian Guo.

**Funding acquisition:** Jian Guo, Li He.

**Investigation:** Zhengzhou Yuan, Muke Zhou, Jian Guo, Yanbo Li, Li He.

**Methodology:** Zhengzhou Yuan, Muke Zhou, Jian Guo, Yanan Zhang, Yanbo Li, Li He.

**Project administration:** Muke Zhou.

**Resources:** Muke Zhou, Yanan Zhang, Li He.

**Software:** Ning Chen, Yanan Zhang, Yanbo Li.

**Supervision:** Yanan Zhang.

**Validation:** Ning Chen.

**Visualization:** Ning Chen.

**Writing – original draft:** Zhengzhou Yuan.

**Writing – review and editing:** Ning Chen, Jian Guo, Li He.

## Supplementary Material

Supplemental Digital Content
