# Supplementary material for: Effects of hypertension in patients receiving mechanical thrombectomy: A meta-analysis
Source: Medicine (Baltimore). 2020 Apr 17;99(16):e19803. doi: 10.1097/MD.0000000000019803 (PMC7440350; doi:10.1097/MD.0000000000019803)
Supplement: Supplemental Digital Content [file medi-99-e19803-s001.docx]

SUPPLEMENTAL

Table 1.

Summary of included articles assessing impact of hypertension and/or admission blood pressure on acute ischemic stroke outcomes after mechanical thrombectomy

| First author | Study type | Study period | Region(s) included | Patients | men | HP# | Age | Thrombectomy device | Embolized site* | NIHSS | Indicators included in the analysis | NOS |
| --- | --- | --- | --- | --- | --- | --- | --- | --- | --- | --- | --- | --- |
| Abilleira | Post analysis of SONIIA registry | January 2011 to December 2012 | Spain, multicenter | 536 | 294 | 321 | 67.5±13.4 | Not specified | A and P | 17.5 (13-21) | hypertension for functional outcome | 8 |
| Abou-Chebl | Post analysis of NASA Registry | March 2012 to February 2013 | USA, multicenter | 354 | 178 | 271 | 67.3±15.2 | Solitaire FR | A and P | 18.1±6.6 | hypertension for functional outcome | 7 |
| Alawieh | Case control | January 2013 to November 2017, | USA, single center | 336 | 159 | 270 | 74.6±8.9 | ACE, Penumbra | A and P | 16.3±7.5 | hypertension for functional outcome | 5 |
| Di | Case control | April 2016 to July 2017 | Italy, single center | 71 | 26 | 45 | 75(45-99) | Not specified | A | 18 | hypertension for functional outcome | 6 |
| Duan | Post analysis of ACTUAL Investigators | January 2014 to June 2016 | China, multicenter | 616 | 368 | 401 | 66 (57–74) | stent retriever | A | 16 (12–21) | hypertension for sICH | 7 |
| Gilberti | Case control | August 2012 to April 2016 | Italy, multicenter | 68 | 34 | 49 | 74 (66–79) | stent retrievers or thromboaspiration | A | 17 (14–21) | hypertension for functional outcome | 4 |
| Gordon | Case control | August 2012 to March 2016 | USA, multicenter | 79 | 39 | 63 | 69.2±13.8 | Trevo, Solitaire, Penumbra | A and P | 16.4±6.3 | hypertension for functional outcome | 5 |
| Goyal | Case control | July 2013 to December 2016 | USA, single center | 88 | 42 | 69 | 62±15 | stent retriever or aspiration | A and P | 16 (12–21) | blood pressure for functional outcome | 5 |
| Goyal | Case control | January 2012 to June 2016. | USA, Single center | 293 | 147 | 231 | 62±14 | stent retriever or aspiration | A and P | 16 (13–19) | hypertension for functional outcome, hypertension for sICH | 6 |
| Imahori | Case control | April 2015 to January 2017 | Japan, single center | 91 | 43 | 52 | 79 (73-85) | stent retriever | A | 15 (8-22) | hypertension for functional outcome | 5 |
| John | Case control | January 2008 to December 2012 | USA, single center | 147 | 66 | 97 | 67±16 | Not specified | A | 15.8 ± 6.7 | blood pressure for functional outcome | 6 |
| Kang | Case control | January 2011 toDecember 2016, | Korea, multicenter | 140 | 91 | 92 | 67(30-88) | stent retriever or manual aspiration | A and P | 13 | hypertension for functional outcome | 4 |
| Linfante | Case control | March 2012 to February 2013 | USA, multicenter | 234 | 114 | 174 | 66.9±14.7 | Solitaire FR | A and P | - | hypertension for functional outcome | 6 |
| Löwhagen | Case control | 2007 to 2012 | Sweden, single center | 108 | 66 | 48 | 70 (62–77) | Not specified | A | 21 (18–24) | hypertension for functional outcome | 5 |
| Maier,I,L | Case control | January 2013 to January 2017 | Germany, single center | 168 | 71 | 133 | 74(61-83) | aspiration catheters with or without retrievable stents | A | 15.1±5.4 | hypertension for functional outcome, blood pressure for functional outcome | 6 |
| Maier,B | Case control | January 2012 to June 2016 | French, multicenter | 1332 | 538 | 587 | 67.6±15.0 | Not specified | A and P | 16 (11-21) | hypertension for functional outcome, hypertension for sICH | 6 |
| Mistry | Case control | March 2015 to October 2016 | USA, multicenter | 228 | 104 | 166 | 65.8 (14.3) | Not specified | A | 16.3±7.1 | hypertension for sICH | 5 |
| Mokin | Case control | March 2012 to March 2016 | USA, single center | 117 | 67 | 88 | 67.0±14.5 | stent retriever | A | 15 | hypertension for functional outcome | 6 |
| Ozdemir | Case control | January, 2011 to February, 2014, | Turkey, multicenter | 70 | 41 | 42 | 57.4 (10.4) | stent retriever | A and P | 20 (18–22) | hypertension for functional outcome | 7 |
| Parrilla | Case control | April 2010 to June 2012 | Spain, single center | 150 | 78 | 106 | - | stent retriever | A and P | - | hypertension for functional outcome | 6 |
| Pikija | Case control | January 2012 to December 2016 | Austria, single center | 164 | 73 | 106 | 74 (20–92) | stent retriever | A | 18 (3–32) | hypertension for functional outcome | 5 |
| Protto | Case control | January 2013 to December 2014 | Finland, single center | 105 | 60 | 46 | 66±11 | stent retriever | A | 14.5 ±5) | hypertension for functional outcome | 4 |
| Psychogios | Case control | - | Germany, single center | 51 | 28 | 44 | 69(21-86) | Penumbra | A | 17.5 ±6 | hypertension for functional outcome | 6 |
| Sun | Case control | December 2010 to March 2013. | USA, multicenter | 106 | 60 | 72 | 66±14 | Not specified | A and P | 19 (15–23) | hypertension for functional outcome | 6 |
| Tajima | Case control | July 2014 to November 2016 | Japan, single center | 69 | 40 | 36 | 74.6±9.2 | Penumbra, stent retrieven | A and P | 20.3±5.7 | hypertension for functional outcome | 5 |
| Tateishi | Case control | June 2010 to May 2011 | USA, single center | 35 | 18 | 24 | 70(57–78) | MERCI device, Penumbra | A | 13 (9–20) | hypertension for functional outcome | 7 |
| Todo | Case control | June 2006 to January 2016 | Japan, single center | 117 | 48 | 71 | 74(65-82) | Merci, Penumbra, stent retriever | A | 18(11-23) | hypertension for functional outcome | 6 |
| Tsogkas | Case control | January 2011 to December 2014 | Germany, single center | 65 | 46 | 57 | 72 (68-76) | Penumbra, stent retriever | A | 17 (12-21) | hypertension for functional outcome | 5 |
| Whalin | Case control | September 2010 to April 2015 | USA, single center | 256 | 123 | 179 | 65.2±15.4 | Not specified | A | 17 (13–22) | hypertension for functional outcome | 6 |
| Yamamoto | Case control | April 2011 to December 2014 | Japan, single center | 76 | 46 | 37 | 76±11 | Not specified | A | 13(4-40) | hypertension for functional outcome | 6 |
| Yoon | Case control | December 2010 to November 2015 | Korea, single center | 335 | 166 | 197 | 72 (64-79) | stent retriever | A | 13 (10-16) | hypertension for functional outcome | 7 |

Abbreviation: HP#: hypertension. Embolized site*: A= anterior circulation; P=posterior circulation. NOS: Newcastlee-Ottawa Quality Assessment Scale.
